# Regulation of mitochondrial functions by protein phosphorylation and dephosphorylation

**DOI:** 10.1186/s13578-016-0089-3

**Published:** 2016-04-14

**Authors:** Sangbin Lim, Kelly R. Smith, Ssang-Taek Steve Lim, Rong Tian, Jianrong Lu, Ming Tan

**Affiliations:** Center for Cell Death and Metabolism, Mitchell Cancer Institute, University of South Alabama, Mobile, AL USA; Department of Biochemistry and Molecular Biology, College of Medicine, University of South Alabama, Mobile, AL USA; Mitochondria and Metabolism Center, Department of Anesthesiology and Pain Medicine, University of Washington, Seattle, WA USA; Department of Biochemistry and Molecular Biology, University of Florida College of Medicine, Gainesville, FL USA

**Keywords:** Mitochondria, Kinase, Phosphatase, Phosphorylation, Metabolism

## Abstract

The mitochondria are double membrane-bound organelles found in most eukaryotic cells. They generate most of the cell’s energy supply of adenosine triphosphate (ATP). Protein phosphorylation and dephosphorylation are critical mechanisms in the regulation of cell signaling networks and are essential for almost all the cellular functions. For many decades, mitochondria were considered autonomous organelles merely functioning to generate energy for cells to survive and proliferate, and were thought to be independent of the cellular signaling networks. Consequently, phosphorylation and dephosphorylation processes of mitochondrial kinases and phosphatases were largely neglected. However, evidence accumulated in recent years on mitochondria-localized kinases/phosphatases has changed this longstanding view. Mitochondria are increasingly recognized as a hub for cell signaling, and many kinases and phosphatases have been reported to localize in mitochondria and play important functions. However, the strength of the evidence on mitochondrial localization and the activities of the reported kinases and phosphatases vary greatly, and the detailed mechanisms on how these kinases/phosphatases translocate to mitochondria, their subsequent function, and the physiological and pathological implications of their localization are still poorly understood. Here, we provide an updated perspective on the recent advancement in this area, with an emphasis on the implications of mitochondrial kinases/phosphatases in cancer and several other diseases.

## Background

The mitochondria are double membrane-bound organelles found in most eukaryotic cells. They generate most of the cell’s energy supply of adenosine triphosphate (ATP). Protein phosphorylation/dephosphorylation is a crucial regulatory system of signal transduction which controls many aspects of cellular functions. It can greatly impact the properties of a protein’s enzymatic activity, structure, subcellular localization and stability. Phosphorylation, accomplished via a kinase action, and its reverse-de-phosphorylation via a phosphatase are essential switching-on and -off mechanisms in cell signaling. Many kinases and phosphatases have been well-documented to play roles in the cell primarily outside mitochondria. Although the function and mechanisms of translocation to mitochondria are not well understood, the evolution of imaging and molecular techniques has allowed the discovery that an increasing number of kinases and phophatases localize to mitochondria. The importance of phosphorylation/dephosphorylation in the governing of mitochondrial processes is supported by recent evidence that mitochondria contain many protein kinases, phosphatases, and associated proteins.

The aim of this review is to provide an updated perspective on the kinases and phosphatases localized to mitochondria. We describe here kinases and phosphatases that associate with mitochondria, their function within mitochondria, identified thus far, and the possible physiological or pathological implications of their mitochondrial localization.

## Kinases localized in mitochondria

### Tyrosine kinases

#### Abl

Abl is a non-receptor tyrosine kinase and is ubiquitously expressed in several cell types and tissues. Abl is localized at distinct sub-cellular sites including the nucleus, cytoplasm and endoplasmic reticulum (ER) and has various binding partners including signaling adaptors, proliferation regulators, transcription factors, kinases, phosphatases and cytoskeletal proteins [1]. While Abl is primarily localized to the nucleus, ER, and cytoplasm, it has been found that about 4 % of the total Abl resides in the mitochondria of murine embryo fibroblasts [2]. It has been revealed that H_2_O_2_ treatment of various cell types causes translocation of Abl to mitochondria and then induces cell death. Apart from H_2_O_2_, stimuli of ER stress, calcium ionophore A23187 and brefeldin A, also promote the translocation of Abl to mitochondria [3]. Although Abl does not contain a typical mitochondrial localization signal of its own, a study showed that protein kinase Cδ binds to Abl in the ER. This PKCδ-Abl complex translocates from ER to mitochondria and then triggers apoptosis [4].

Abl contributes to human diseases such as Alzheimer’s disease (AD) and chronic leukemia. Exposure of cultured neurons to various forms of multimeric amyloid-β peptide led to an increase in Abl tyrosine kinase activity and subsequent tyrosine phosphorylation of tau at Y394 [5–7]. Additionally, it was found that abnormal tau phosphorylation and subsequent cell death could be prevented by the Abl family kinase inhibitor [6, 7].

In chronic myeloid leukemia (CML) and acute promyelocytic leukemia (AML), Abl is activated upon translocation within breakpoint cluster region (Bcr) gene [8, 12]. Bcr/Abl fusion protein is a constitutively active tyrosine kinase which allows cells to proliferate without cytokine regulation, leading to a clonal myeloproliferative disorder. Its inhibitor, imatinib mesylate, can trigger apoptosis of CML cells [9]. It will be intriguing to explore if Abl or Bcr/Abl-associated apoptosis in AD and chronic leukemia is directed by mitochondrial localized Abl activity.

### Src family kinases

There are multiple lines of evidence support the mitochondrial localization and function of Src family kinases. Although Src family kinases are permanent residents of cytoplasm, a previous study has suggested Src family including Src, Fyn, Lyn, Fgr and a negative regulator of Src family kinases, CSK, can be localized in mitochondria [10]. The phosphotyrosine signal in mitochondria was dramatically decreased by pretreatment with a Src inhibitor, PP2. This result suggests that Src family kinases may be associated with mitochondrial protein phosphorylation. Other groups performed subfractionation of mitochondria using proteinase K and/or Triton X-100, further supported the localization of Src family kinases within mitochondria [11].

Since Src family kinases do not contain typical mitochondrial localization signals, they seem to be dependent on unidentified adaptor proteins for translocation into mitochondria. Several studies revealed that two anchoring proteins of protein kinase A (PKA), A Kinase Anchor Proteins 121 (AKAP121) and Dok-4, associate with Src. It is well known that AKAP121 acts as a multifunctional protein binding to protein tyrosine phosphatase D1 (PTPD1). PTPD1 anchors Src to the outer membrane of mitochondria upon activating the protein [12, 13]. Interestingly, a study showed that AKAP121 is found in the mitochondrial inner membrane with Src and Lyn [14]. Dok-4 is an adapter protein and appears to be responsible for the mitochondrial importation of Src in bovine endothelial cells. While deficiency of Dok-4 induces Src localization to extra-mitochondria, overexpression of Dok-4 causes Src localization to mitochondria [15].

Mitochondrial electron transport chain (ETC) is the final component of aerobic respiration, which contains a series of electron transporters embedded in the inner mitochondrial membrane. It includes complex I to IV and creates a chemical gradient that allows for the production of ATP. Several ETC complexes have been identified as substrates for Src. The first protein shown to be activated by mitochondrial Src was cytochrome c oxidase (complex IV in ETC) [11]. Src phosphorylates a subunit of cytochrome c oxidase, and leads to the enzymatic activation of complex IV in osteoclasts. Recently, it has been found that Src also affects other complexes within the ETC. One study showed that an increase in Src activity, as a response to changes in ATP levels in mitochondria of the rat brain, leads to an increase in the activities of complexes I, III, and IV, along with decreases in activity of complex V [16]. Two groups reported that inhibition of Src activity led to reduction in mitochondrial respiration mediated by a specific decrease in actions of complex I’s NADH dehydrogenase-ubiquinone oxidoreductase system. NADH ubiquinone flavoprotein 2 at Tyr 193 of complex I and succinate dehydrogenase A at Tyr 215 of complex II, are the targets of Src phosphorylation [17, 18].

c-Src, normal proto-oncogene Src, possesses anti-apoptotic properties and shows increased protein levels and activity in a variety of human cancers [19]. Further, c-Src has been found to be a crucial player in multiple signaling pathways regulating proliferation, survival, metastasis, and angiogenesis [19]. One of these pathways is that of the mitochondrial, hypoxia-mediated ROS-induced activation of c-Src, HIF-1α, and NF-κB which contributes to cell survival in various cancers including hepatomas, colon carcinomas, and neuroblastomas [20]. Additionally, c-Src has an important role in serum deprived pancreatic cancer cell survival [21]. Also, c-Src leads to metastasis of diverse cancers including breast cancer [22].

Mitochondrial Fgr kinase regulates complex II activity by tyrosine phosphorylation and plays an important role in regulating NADH/FADH_2_ ratio. H_2_O_2_ promotes Fgr localization to mitochondria, but it does not seem that Fgr has a consensus signal sequence for mitochondrial localization [23].

Hibbs et al. found that mice homozygous for Lyn locus aberrations show abnormalities connected to B lymphocyte lineage and mast cell function [24]. Lyn-/- mice did not mediate an allergic response to IgE cross-linking and exhibited severe glomerulonephritis caused by the kidney deposition of IgG immune complexes [24, 25], indicating that Lyn is associated with autoimmune disease. Lyn was also discovered to be critical for maintenance of the leukemic phenotypes of many different hematopoietic cancers including AML, CML and B cell lymphocytic leukemia [26–28]. Lyn was also expressed in some solid tumors. Therefore, it could serve as a potential therapeutic target for prostate cancer, glioblastomas, and aggressive subtypes of breast cancer [29–31]. Recent study revealed that Lyn-mediated mitochondrial tyrosine phosphorylation is required for hepatocyte survival under partial hepatectomy, pro-apoptotic conditions [32]. However, the role of Lyn in mitochondria and human diseases needs to be further elucidated.

### Receptor tyrosine kinases

#### EGFR

A recent study demonstrated that epidermal growth factor receptor (EGFR) is localized to mitochondria where it interacts with cytochrome c oxidase subunit II (CoxII) in mouse fibroblasts over-expressing EGFR and Src [33]. EGF stimulation promotes translocation of EGFR into mitochondria. The translocation depends upon the phosphorylation of EGFR on Y845 by Src. The translocation is not observed upon overexpression of a catalytically inactive Src mutant or upon replacement of Y845 by phenylalanine (Y845-EGFR). A similar study showed that CoxII was phosphorylated by Src and EGFR, and the EGFR’s mitochondrial localization was increased by EGF stimulation. Mitochondrial EGFR decreased the activity of Cox and reduced cellular ATP, indicating that mitochondrial EGFR regulates the function of mitochondria [34]. Moreover, new evidence showed that EGFR and EGFRVIII, a constitutively active variant of EGFR were localized in mitochondria [35, 36]. EGFR co-localized with FAK and Src in the mitochondria in glioma and xenografts. This suggests a role of mitochondrial EGFR in cancer progression [37].

There is a debate as to whether the translocation of EGFR depends upon its endocytosis. One group concluded that the EGFR’s localization to mitochondria depends on its endocytosis [38]. The report revealed that EGFR’s mitochondrial translocation can be dramatically decreased in the presence of 3-methyladenine (3-MA), an autophagy inhibitor. Also, the knockdown of Beclin 1, an autophagy related gene, markedly decreased the mitochondrial translocation of EGFR. However, another group suggested that mitochondria-localized EGFR is independent of its internalization [39]. Therefore, the detailed mechanisms of EGFR mitochondrial localization still remain unclear.

#### ErbB2

ErbB2 is a human version of EGFR2. Recent study revealed that ErbB2 localizes to mitochondria of breast cancer cells and tumor samples of patients [40]. The study also found that localization of ErbB2 into mitochondria is mediated via association with mtHSP70 and reduces mitochondrial respiratory functions, including oxygen consumption, while it enhanced cellular glycolysis. It also showed that mitochondrial ErbB2 decreased membrane potential as well as the cellular ATP levels. However, detailed molecular mechanisms or direct substrates of mitochondrial EGFR and ErbB2 remain unclear. It is well known that EGFR and ErbB2 have been associated with the development of numerous human cancers. Tumors with changes in EGFRs tend to be more aggressive, and are considered as indicators of a poor clinical outcome, therefore they are intensely studied as therapeutic targets [41].

### Serine/threonine kinases

#### Akt

It is well known that activated protein kinase B (Akt) is localized to diverse subcellular compartments: these include the Golgi, endoplasmic reticulum, and the nucleus. Also, it was shown that Akt could be localized in mitochondria when stimulated by 17β-estradiol and insulin in endothelial cells [42]. In human neuroblastoma, it was reported that the activation of PI3 K/Akt signaling via insulin or insulin-like growth factor-1, is greatly amplified by mitochondrial Akt locolization [43]. Recent studies also showed that treatment with leukemia inhibitory factor (LIF) increased the amount of total and phospho-Ser473-Akt in mitochondria [44], and insulin treatment could translocate Akt to mitochondria in cardiac muscle cells [45].

Several studies reported that mitochondrial Akt causes phospho-inactivation of the pro-apoptotic protein BAD, and recruitment of Raf-1 to the mitochondria promoted cell survival [46, 47]. Recent studies also discovered new targets for mitochondrial Akt such as mitochondrial electron complex V and hexokinase-II [44, 45]. Akt isoform 1 modulates mitochondrial complex V activity, enhances the production of ATP, and increases phosphocreatine in cardiomyocytes, indicating that Akt is associated with ATP metabolism [45]. Akt also has a direct effect in mitochondria, which is mediated by phosphorylation of hexokinase-II, resulting in protection of the mitochondria from oxidant or Ca^2+^-induced mitochondrial permeability transition pore (MPTP) opening [44]. Yang et al. found that translocation of phospho-Akt to mitochondria was enhanced in the streptozotocin-induced diabetic mice and insulin stimulates translocation of Akt to mitochondria [48]. Barksdale et al. revealed that HSP90 is responsible for Akt accumulation in mitochondria in unstimulated cells [49]. Taken together, mitochondrial Akt may play important roles in energy metabolism associated with diabetes.

#### JNK

It was well established that the c-Jun N-terminal kinase (JNK) localizes in cytoplasm and the nucleus. Many studies show that JNK may be activated in or translocated to the mitochondria. It was first revealed that JNK is localized in mitochondria in the phorbol ester response of myeloid leukemia cells [50]. Recent studies suggested an important role of JNK in mitochondria-related functions. These studies include murine heart mitochondria, hydrogen peroxide-treated rat brain or primary cortical cultures, acetaminophen-induced liver damage, multiple myeloma cells treated with anti-cancer drugs, and neonatal ischemia [51–57].

As mentioned before, JNK regulates apoptosis in general, indicating that targets of JNK can be apoptosis related proteins. JNK mediates phosphorylation and oligomerization of proapoptotic BAD, initiating apoptosis [58]. Conversely, there are studies reporting that JNK reduces apoptosis. For example, JNK is necessary for IL-3-mediated cell survival via phosphorylation and inactivation of BAD [59]. Also, activated JNK may co-localize with, and phosphorylate, Bcl-2 in mitochondrial membranes of hematopoietic cells [60]. This effect is present during interleukin-3-mediated stress, resulting in enhanced anti-apoptotic functions of Bcl-2. These contradictory results indicate that the localization and function of JNK in mitochondria are not well understood and warrant further investigation.

#### ERK1/2

Although extracellular signal-regulated kinase 1 and 2 (ERK1/2) normally translocates between cytosol and the nucleus to influence trophic and pro-survival functions, recent studies found that ERK1/2 can localize to mitochondria of mouse heart [51], renal epithelial cells [61], mouse hippocampus [53] and human alveolar macrophages [62]. Mitochondrial ERK1/2 appears to play a crucial part in mitochondrial function [61, 62] including mitochondrial dysfunction, mitophagy, and apoptosis [63–65]. Recent investigation found that mitochondrial ERK1/2 phosphorylates steroidogenic acute regulatory protein (StAR), and this phoshporylation by ERK is required for the maintenance of this protein in mitochondria. Mitochondrial StAR together with mitochondrial active ERK and PKA are necessary for maximal steroid production [66].

#### p38 MAPK

Several studies revealed that the p38 mitogen-activated protein kinase (p38 MAPK) could reside in mitochondria. One group, using immunoblotting and immunofluorescence, showed that p38 MAPK is localized in cardiac mitochondria [51]. p38 MAPK was activated in the mitochondrial fraction by ischemia [67] and increased in mitochondria under H_2_O_2_ treatment [68]. Although it is well known that p38 MAPK performs a crucial function in mitochondria-mediated apoptosis, the precise function of mitochondrial p38 MAPK remains to be elucidated.

#### GSK3β

It has been established that glycogen synthase kinase (GSK) 3β can be localized in cytosol and nucleus. Some substrates of GSK3β (e.g., tau), are cytosolic, whereas others (many transcription factors) are nuclear. However, in 1995, it was revealed that GSK3β is present in mitochondria in rat cerebellum [69]. Since then several studies showed that GSK3β regulates the function of mitochondrial proteins such as adenine nucleotide translocator (ANT) and cyclophilin D [70–72]. The mechanism of GSK3β’s translocation to the mitochondria is unclear, but TOM20 may be integral, as in the example of connexin-43 [73]. In mitochondria, GSK3β phosphorylates and suppresses a critical mitochondrial enzyme, pyruvate dehydrogenase activity [74]. Also, it has been demonstrated that GSK3β has a role in determining the threshold for mitochondrial permeability transition pore (MPTP) opening [75], indicating that GSK3β regulates mitochondria-mediated apoptosis.

Accumulated evidence shows that GSK3β is highly associated with neurodegenerative diseases. GSK3 is a key kinase contributing to aberrant phosphorylation of the microtubule-binding protein tau in a process thought to cause neurofibrillary tangles in Alzheimer’s disease [76]. In 1994, Mulot et al. found, via SDS-PAGE, that paired helical filament-tau from the AD brain comprises four species which can be mimicked by GSK3β mediated human brain tau phosphorylation in vitro [77]. Recent study also revealed that, in primary cortical neurons, GSK3β is involved in Amyloid-β phosphorylation [78]. GSK3β polymorphism was found to be associated with Parkinson’s disease (PD) that alters GSK3β transcription and splicing [79].

There is much interest in GSK3β as a potential therapeutic target in many types of cancer. However, the application is complicated by findings that GSK3β behaves as a tumor suppressor, but may promote cell proliferation in different types of cancer. There is evidence that GSK3β functions as a tumor suppressor in skin and breast cancer [80, 81] and that repression of RNA polymerase 1 transcription by GSK3β contributes to this tumor suppressor action [82]. However, there are contradictory findings that GSK3β mediates tumor promotion and/or GSK3β shows anti-proliferative effects in certain types of tumors including colon and pancreatic cancer [83, 84]. Whether these appeared contradictory functions of GSK3β can be explained by the different localizations of this protein? It is still not clear if mitochondrial GSK3β is associated with neurodegerative diseases or cancer.

#### PKA

It has been acknowledged for many years that Protein kinase A (PKA) is crucial to mammalian mitochondrial physiology. Since the 1970s, it has been shown that PKA associates with, and in, mitochondria [85–88]. While catalytic subunits of PKA are predominantly found in the mitochondrial outer membrane [89], there is much evidence that PKA localizes to the mitochondrial inner membrane and matrix as well [87, 90–93]. In general, PKA is concentrated in cellular membranes and organelles through interactions with A kinase anchor proteins (AKAPs) [94, 95]. One group showed that de-localization of PKA from mitochondria was prompted by dominant negative AKAP121 mutation [96]. Indeed, expression of AKAP121 targets PKA to the cytoplasmic surface of mitochondria and then refines cAMP-PKA signaling to mitochondria [97]. Recent investigation revealed that AKAP1 recruits PKA and other signaling proteins to the outer mitochondrial membrane and thereby integrates several second messenger cascades by inhibitory phosphorylation of dynamin-related protein 1 (Drp1) and maintenance of mitochondrial integrity [98].

Drp1 is a mechanoenzyme using GTP hydrolysis to fuel the division of mitochondria. PKA mediated phosphorylation of Ser637 on Drp1 blocks Drpl’s translocation to the mitochondrial surface and influences on fission [99]. Other findings indicate that outer mitochondrial PKA, and phosphatase PP2A, regulate neuronal development by inhibiting and promoting mitochondrial division [100].

The cAMP response element-binding protein (CREB) is a transcription factor which regulates the transcription of cAMP response element-regulated genes and is found to bind to the mitochondrial DNA [101]. It has been shown that CREB is involved in regulation of mitochondrial gene expression and neuron longevity. Interestingly, it has been suggested that CREB may be phosphorylated by PKA within mitochondria [102].

Many heat shock proteins (Hsps) are known to play essential protective roles in the cardiovascular system. Hsp20 is expressed at high levels in cardiac, skeletal, and vascular smooth muscle. Hsp20 is regulated by the β-adrenergic/cAMP/PKA signaling pathway, which is known to be chronically activated in heart failure [103]. A recent study also revealed that PKA’s phosphorylating Ser16 of Hsp20 is vital to the small Hsp’s cardioprotective action [104]. For therapeutic development, it will be critical to distinguish if PKA mediated cardiprotective effects are due to mitochondrial PKA activity.

#### PKC

Protein kinase C (PKC) is a group of phospholipid-dependent serine/threonine kinases regulating numerous cellular functions through key signaling molecules. The activated PKCs translocate to multiple subcellular sites. Interestingly, analysis of the subcellular distribution of PKCε in mouse heart with constitutively active PKCε revealed that activated PKCε is associated with a variety of mitochondrial proteins [51]. It has been suggested that mitochondrial translocation of PKCε is associated with cardioprotection [105], and several studies showed that PKCε substrates reside within cardiac mitochondria [106–108]. COX IV has been identified as a substrate of PKCε in mitochondria [106]. Phosphorylation and activity of COX IV were increased by PKC activator, phorbol 12-myristate 13-acetate (PMA), but were blocked by the selective PKCε inhibitor, εV1-2. Another known mitochondrial target for PKCε is the MPTP [107]. MPTP remains tightly closed under normal conditions, but it opens under cell death conditions. PKCε interacts with presumed components of the MPTP in heart mitochondria, leading to reduced MPTP opening. Another target for PKCε is a mitochondrial ATP-sensitive K+ channel (mitoK_ATP_) [108]. It was recently found that PKC mediates interactions between conexin43 and mitoK_ATP_ subunit in mitochondria of cardiomyocytes [109]. These channels are normally closed, but are opened during periods of metabolic stress as ATP levels decline, leading to channel opening-mediated protective effects. This evidence reveals that mitochondrial PKCε has a pro-survival role in a stress condition.

Hamasaki et al. found that expression of PKCε decreases by 40 % in cardiac ventricles of hyperthyroid rats [110]. The expression of PKCε was reduced in cytosol and membrane fractions but was not reduced in extracts of hypertrophied cardiac ventricles generated by aortocaval shunt or aortic banding. Other groups also revealed that hypertrophic stimuli activated PKCε in rat-cultured cardiac myocytes, and in vivo, and that PKCε overexpression in mice leads to cardiac hypertrophy related to concentric remodeling and the preservation of cardiac contractility [111, 112].

Recent study revealed that PKCɛ increases the activity of endothelin converting enzyme, which degrades amyloid-β, and decreases amyloid plaque in transgenic mice [113], indicating that greater neuronal PKCɛ activation may promote amyloid-β clearance, reducing Alzheimer’s neuropathology. However, it has not been examined whether mitochondrial PKCɛ is needed for AD progression.

PKCδ also translocates to mitochondria. Diverse studies revealed that treatment in a variety of neoplastic cells with hydrogen peroxide, phorbol esters, or anticancer agents such as cisplatin and etoposide causes PKCδ translocation into the mitochondria [114–116]. A study also found that mitochondrial PKCδ triggers the release of cytochrome c and resultant cell death. Thus, overexpressed PKCδ promotes apoptosis in neoplastic and normal keratinocytes by targeting mitochondria and disturbing their membrane potential [117]. However, direct targets for mitochondrial PKCδ still remain unclear.

#### PINK1

The PTEN-induced kinase 1 (PINK1) is a serine/threonine kinase. It is well known that PINK1 is a factor in autosomal recessive familial PD [118]. A large amount of evidence shows that PINK1 is localized in mitochondria and regulates mitochondrial function. A study found a strong mitochondrial targeting signal domain at its N terminus and showed that N-myc-tagged PINK1 expressed in mammalian cells accumulated in mitochondria. The protein could be found on the inner and outer mitochondrial membrane [119, 120]. PINK1 regulates Parkin (a protein related to PD), which acts as an E3 ubiquitin ligase [121]. PINK1 recruits Parkin to depolarized mitochondria. While PINK1 is imported and rapidly degraded by presenilin-associated rhomboid-like serine protease (PARL) in mitochondria with intact membrane potential, PINK1 breakdown is impaired in mitochondria with reduced membrane potential [120]. Apart from Parkin, PINK1 phosphorylates the mitochondrial fusion protein MFN2, which then acts as a mitochondrial receptor for Parkin [122] and Miro, an atypical Rho GTPase that tethers mitochondria to the tubulin network [123]. Several studies showed that the PINK1/Parkin pathway is associated with fission and fusion events of mitochondria. Those studies revealed that the PINK1/Parkin in mitochondria regulates pro-fusion pathway given that the overexpression of PINK1 leads to interconnected and structurally elongated mitochondria, whereas knockdown of PINK1 leads to fragmentation of mitochondria [124, 125].

PINK1/Parkin pathway also regulates transport of mitochondria. Damaged mitochondria, prior to their clearance, are sequestered via the PINK1/Parkin pathway where mitochondrial movement is prevented through PINK1 mediated-Miro phosphorylation [123, 126]. PINK1 regulates mitochondrial content through mechanisms independent of mitophagy and PINK1-knockout mice showed a decreased Complex I activity [127, 128]. PINK1 mutant flies showed complex I deficiency and impaired ATP generation; however, supplementing these PINK1-deficient flies with complex 1 or ubiquinone corrects reductions in ATP generation allowing for recovery of flight muscle [129]. In both PINK1 and Parkin null flies, there lies a widespread defect with turnover of electron transport chain proteins [130]. Recent study demonstrated that PINK1 and Parkin mediate localized translation of respiratory chain component mRNAs along the mitochondrial outer membrane [131].

Dagda et al. studied the relationship between a PINK1 knock down and mitochondrial dysfunction in PD [132]. Stable knockdown of PINK1 produced mitochondrial fragmenting and autophagy in SH-SY5Y cells. Recent study found that loss of PINK1 accelerates neurodegenerative phenotypes induced by mitochondrial stress in transgenic animals where exists the conditional expression of mitochondrial unfolded ornithine transcarbamylase [133]. Mutations associated with PD were found throughout PINK1; however, the majority lay within the kinase domain, suggesting that loss of PINK1 kinase function is part of the pathogenesis [134]. Further, mutations within the carboxy terminus of PINK1 turn out to be critical to control an optimal kinase activity [135].

There is substantial evidence, based on epidemiological studies, for lower rates of cancer in patients with PD. The relationship between cancer incidence and PD may be related to the presence of genes common to both diseases [136]. Investigators found that PTEN knock-down suppresses PINK1 levels and shows cardioprotective effects, and concluded that PINK1 is a potential novel cardioprotective kinase [137]. However, the role of PINK1 in cancer development and progression is unclear. One study reported a link between Type II diabetes and PINK1 [138]. Investigators found that PINK1 was reduced in the skeletal muscle of Type II diabetic patients and in conditions of obesity or inactivity (or both) in patients and controls. One study found glucose transporter inhibition under loss of PINK1 [139]. Recently, Deas et al. reported that the loss of PINK1 function seems to disturb glucose-sensing, resulting in enhanced insulin release, which is uncoupled from glucose uptake in beta cells [140], suggesting that a deficiency of the Parkinson’s-associated PINK1 protein may directly alter insulin secretion at the pancreas.

## Phosphatases translocate into mitochondria

### Protein phosphatases

#### MKP1

MAP Kinase Phosphatases (MKPs) are dual specificity phosphatases which dephosphorylate both pSer/pThr and pTyr. MKP1 is localized in the nucleus through a LXXLL motif and appears to be confined to the dephosphorylation of nuclear MAPKs [141]. However, recent study found that MKP1 translocates to mitochondria after nerve growth factor treatment [142]. This result appears reasonable given that its substrates, and MAPKs, are also localized in mitochondria.

Wang et al. found that, in malignant samples, MKP1 is increased five fold over non-malignant samples [143]. This indicates that therapeutic suppression of MKP1 action may allow for expression of pro-apoptotic signaling of JNK in malignant cells. MKP1 is highly expressed in various human tumors, including non-small-cell lung cancer (NSCLC), bladder, ovarian, breast, osteosarcoma and prostate cancers [144–146]. They also found that MKP1 plays a critical part in the pathology of NSCLC, both in tumor growth and in response to treatment with cisplatin [146]. Recent study demonstrated that interference of MKP1 actions led to diminished invasion potential, tumor growth, and metastasis in mice [147].

A role for MKP1 in modulation of innate immune responses is strongly supported by several recent papers using models of endotoxic shock, anaphylaxis and arthritis. Mice deficient in MKP1 were hypersensitive to endotoxic shock; this was associated with prolonged activation of MAPKs, and enhanced production of TNF-α, IL-6, and IL-10 [148–150]. Moreover, it has been found that the absence of MKP1 markedly exacerbates disease development in mouse model of rheumatoid arthritis (RA). Overall, MKP1 controls dephosphorylation of MAPKs, but it is not clear whether this is the case for mitochondrial MAPKs and whether MKP1 is associated with human diseases due to mitochondrial MKP1 function.

#### Shp2

Src homology 2 domain-containing phosphatase 2 (Shp2) is ubiquitously expressed and contains two N-terminal SH2 domains along with a C-terminal protein tyrosine phosphatase domain. Shp2 was primarily located inside the mitochondria related to cristae and the intercristal space [16, 151]. Recent study also found that sepsis induced Shp2 mitochondrial localization and expression [152]. Mutations of Shp2 give rise to many distinct human diseases: Germ line Shp2 mutations result in Noonan Syndrome (NS), one of the most common autosomal dominant disorders, and LEOPARD Syndrome (LS), known, as its acronym reminds us, for its major manifestations: multiple Lentigines, Electrocardiographic aberrations, Ocular hypertelorism, Pulmonary stenosis, Abnormalities of genitalia, Retardation of growth, and sensorineural Deafness [153, 154]. The distinct pathogenic routes of NS and LS are driven by different Shp2 mutations: NS-associated Shp2 mutations typically alter residues at the interface of N-SH2 and PTP domains [155], leading to increased enzymatic activity and RAS/ERK activation. LS mutations impact residues of the PTP domain, resulting in dramatic reduction of catalytic activity along with reduced activation of RAS/ERK [156].

Somatic Shp2 mutations are found in ∼35 % of patients with sporadic juvenile myelomonocytic leukemia (JMML), a clonal pediatric myeloproliferative disorder (MPD) featuring the amplified expansion and tissue infiltration of myeloid cells, along with macrocytic anemia and persisting fetal hemoglobinemia [157, 158]. LS patients often undergo hypertrophic cardiomyopathy; in addition they may see an elevated potential for neuroblastoma and AML [159, 160]. Although shp2 expression and activity is closely associated with several diseases, connection between mitochondrial shp2 and disease progression has not been well established.

#### PTPs

Several protein tyrosine phosphases (PTPs) such as PTPD1, PTP1B, and PTPMT1 has been reported as mitochondrial localized PTPs. PTPD1 is localized to outer membrane of mitochondria by binding to AKAP121 (or spliced isoform AKAP84) and increase a magnitute of EGF stimulated signaling [13]. In addition, PTPD1 binds to Src and activate Src, enhancing EGF-dependent mitogen signaling, enhancing oxidative phosphorylation and mitochondrial membrane potential [12]. PTP1B also localizes to mitochondria and enhances Src mediated mitochondrial oxidative phosphorylation [161]. Dual phosphatase PTPMT1 (PTP localized to the mitochondrion 1) has N-terminal mitochondrial localization signal sequence and is found in the matrix face. Knock-down of PTPMT1 alters mitochondrial phosphoprotein profile and markedly enhances ATP production [162]. This study clearly demonstrated the importance of protein phosphorylation/dephosphorylation switch in regulating mitochondrial function.

### Lipid phosphatase

#### PTEN

Phosphatase and tensin homolog deleted on chromosome 10 (PTEN) is a well-known lipid phosphatase. Early studies proposed that PTEN localized exclusively to the cytoplasm and was able to transiently associate with the plasma membrane depending on the local PIP2 and PIP3 concentrations [163]. However, PTEN has recently been shown to localize to specialized subcellular compartments, such as the nucleus and the nucleolus, the mitochondria and the endoplasmic reticulum. Zhu et al. observed that a gradual buildup of PTEN in mitochondria occurred after induction of apoptosis, which was accompanied by the translocation of Bax to mitochondria [164]. Further, Zu et al. revealed that ischemia/reperfusion (I/R) induces mitochondrial localization of PTEN in the myocardium. Moreover, ischemic preconditioning attenuates mitochondrial localization of PTEN post-I/R, possibly blocking the translocation of Bax to the mitochondria, and leading to improved cell viability [165]. Liang et al. characterized an N-terminally extended form of PTEN (named PTENα) that localizes to the cytoplasm and mitochondria, and induces cytochrome c oxidase activity and ATP generation in mitochondria [166]. This evidence showed that PTEN could exist in mitochondria, playing a crucial role in cellular functions such as apoptosis. However, whether this function of PTEN depends on its lipid or protein phosphatase activities and what are the substrates in mitochondria are still not clear.

## Molecular machinery for protein import into mitochondria

**Fig. 1:**
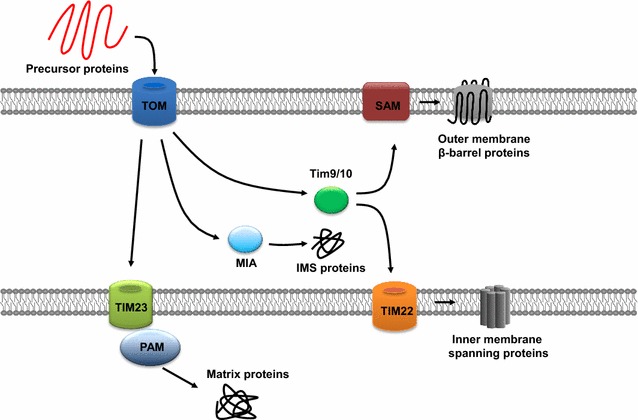
Mitochondrial import pathways. First, nuclear-encoded mitochondrial proteins are imported by the TOM complex. Then, these proteins head to different pathways. Proteins with presequence are transported by the TIM23 complex and the PAM into the matrix. The intermembrane space localized proteins are imported via the MIA. β-barrel precursors of the outer membrane are transferred by the Tim9–Tim10 complex from TOM to SAM. Precursors of inner membrane carriers are inserted by the TIM22 complex into the inner membrane

The majority of mitochondrial proteins are encoded in the nucleus, synthesized in the cytosol, and then delivered into their proper organelle. These mitochondrial proteins are imported into one of four mitochondrial compartments: outer membrane, intermembrane space, inner membrane, and matrix (Fig. 1). Each compartment contains translocases, which interact with precursor proteins to regulate their transport. The major molecular machinery which translocates proteins across the mitochondrial outer membrane is TOM (translocase of the outer membrane of mitochondria) complex. TOM comprises numerous integral membrane protein components: receptor subunits including Tom70 and Tom20, and core translocase subunits including Tom5, Tom6, Tom7, Tom22, and Tom40 [167, 168]. Upon transiting the channel of the TOM complex, substrate proteins may interact with one of three distinct machineries: translocase of inner mitochondrial membrane (TIM) complex 23, Tim9-Tim10 chaperone complex, and mitochondrial intermembrane space assembly machinery (MIA). The classic mitochondrial protein import pathway involves N-terminal presequences on the precursor proteins. These proteins, following passage through the TOM complex, are directed to TIM23 complex and TIM23 bound presequence translocase-associated motor (PAM) which completes pre-protein translocation into the matrix. Following this, mitochondrial processing peptidases remove the presequences, and the proteins are folded to their native conformations. However, many mitochondrial precursor proteins are translated without cleavable presequences. These proteins also can be imported into mitochondria. The precursors of outer membrane beta-barrel proteins are transported, via the Tim9-Tim10 chaperone complex, to the sorting and assembly machinery (SAM) complex of the outer membrane [169]. Spanning proteins of the inner membrane also interact with the Tim9-Tim10 chaperone complex, after which they are inserted into the inner membrane by the TIM22 complex [170]. Several proteins of the intermembrane space contain cysteine motifs and are imported and oxidized by MIA [171].

As far, the detailed information of the mechanism of translocation of the kinases and phosphatases to mitochondria are elusive. The majority of the reported mitochondrial localized kinases/phosphatases lack typical mitochondrial localization signal. This raises three possibilities. First, it does not need a typical mitochondrial localization signal to be localized in mitochondria; second, there are hidden undiscovered mitochondrial localization signal; third, it is passively transported to mitochondria by a cargo carrier. Since it has been shown that mitochondrial kinases/phosphatases play important roles in regulating various cellular functions, more investigations on the mechanism of the importation are definitely needed.

## Concluding remarks and future directions

**Fig. 2:**
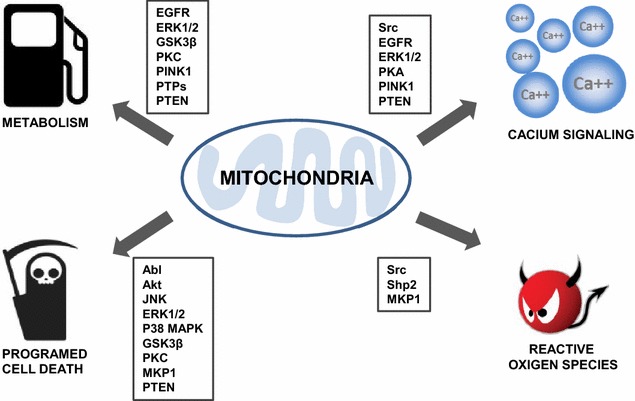
Schematic depiction of the functions of mitochondrial kinases/phosphatases

This review summarizes recent findings concerning mitochondrial localization of kinases and phosphatases, recaps our knowledge on their function to cellular domains beyond those commonly cited in textbooks (Table 1). Over the past many years, the role of mitochondria has been underestimated and understudied. Evidence revealed by recent advanced laboratory techniques such as tandem mass spectrometry, confocal imaging, and electron microscopy, reevaluate mitochondria as a crucial platform for cellular signaling beyond just energy production in the cell. Mitochondria have diverse reported phosphoproteins, kinases and phosphatases, as we have described here. Mounting evidence support that phosphorylation or dephosphorylation of mitochondrial proteins influences mitochondrial function, including metabolism of sugar, amino acids, and lipids; oxidative phosphorylation, antioxidant protein expression, mitochondrial fission and fusion, and decision for survival/death. Emerging evidence has shown that mitochondrial kinases/phosphatases regulate a variety of key regulatory processes in diverse mammalian tissue [172–175]. Intriguingly, a recent phosphoproteome analysis of functional mitochondria showed there are 155 phosphorylation sites in 77 mitochondrial proteins including inner membrane ETS and enzymes in resting human muscle [176]. Although the detailed functions of these kinases/phosphatases in mitochondria are still unclear, there is evidence that the translocated mitochondrial kinases/phosphatases may be seen in mitochondria in several mitochondrial compartments: Those in the matrix likely regulate enzymes of the TCA cycle, amino acid metabolism, free-radical balance, ETC activity, and ATP synthesis (Fig. 2). Those in the outer membrane regulate transport, cell death-related proteins, and mitochondrial fission/fusion. Those at the inner membrane regulate free-radical balance, nucleotide transport, ETC activity and complex assembly.

**Table 1:** Mitochondrial kinases/phosphatases

Proteins	Evidences of mitochondria localization	Functions in mitochondria	References
Abl	Co-IP, WB, IF	Apoptosis	[3, 4]
Src	WB, IP, Co-IP, Kinase array	Cytochrome C oxidase, ROS production and respiration	[10, 11, 15–18]
EGFR	WB, IP, IF	Apoptosis, respiration and cellular metabolism	[33–40]
Akt	WB, IP, Co-IP, IF	Cell survival and regulation of respiration	[43–45, 47–49]
JNK	WB, IF	Apoptosis, neuro inflammation and mitochondria biogenesis Possibly regulation of signal transduction	[50–57, 60]
ERK1/2	WB, IF	Respiration, ATP production, membrane potential, mitophagy, autophagic cell death, MPTP and steroidogenesis	[51, 53, 61–66]
P38 MAPK	WB, IF, GST	Apoptosis	[51, 67, 68]
GSK3β	WB, Co-IP	MPTP and energy metabolism	[70–72, 74, 75]
PKA	WB, IF, DEAE, GST, in vitro kinase assay	Respiration, mitochondria division and steroidogenesis	[87, 89– 93, 95, 98, 99, 102]
PKC	WB, IP, IF	Respiration, K + channel and apoptosis	[105–109]
PINK1	WB, IF	Mitochondria trafficking at outer membrane. Pathogenesis of PD. Localized translation of respiration chain complex	[121, 122, 125–128, 130, 131]
MKP1	WB, IF	Apoptosis	[142]
Shp2	WB, Co-IP	ROS production	[16, 151, 152]
PTPs	WB, Co-IP, IF	ATP production	[12, 13, 161, 162]
PTEN	WB, IF	Apoptosis and ATP production	[164, 165]

*WB* western blot, *Co*-*IP* Co-immunoprecipitation, *IP* Immunoprecipitation, *IF* Immunofluorescence, *GST* GST pull-down assay, *DEAE* diethylaminoethyl cellulose chromatography

In summary, the research community has accumulated a large amount of evidence to support that phosphorylation and dephosphorylation of mitochondrial proteins influence mitochondrial function. However, the strength of the evidence on mitochondrial localization and their activities of the reported kinases and phosphatases vary greatly, and the detailed mechanisms on how these kinases/phosphatases translocate to mitochondria and the physiological and pathological roles related to their mitochondrial localization are still poorly understood. Due to increasing evidence supporting the important functions of mitochondrial kinases/phosphatases, mitochondrial biology is due for more intense exploration in this area.

## Abbreviations

ER: endoplasmic reticulum
AD: Alzheimer’s disease
CML: chronic myeloid leukemia
BCR: breakpoint cluster region
AML: acute promyelocytic leukemia
PKA: protein kinase A
AKAP121: a kinase anchor proteins 121
PTPD1: protein tyrosine phosphatase D1
ETC: mitochondrial electron transport chain
EGFRs: epidermal growth factor receptors
CoxII: cytochrome c oxidase subunit II
3-MA: 3-methyladenine
SH: secondary hyper-parathyroidism
Akt: protein kinase B
LIF: leukemia inhibitory factor
MPTP: mitochondrial permeability transition pore
JNK: c-Jun N-terminal kinase
ERK1/2: extracellular signal-regulated kinase 1 and 2
p38 MAPKs: p38 mitogen-activated protein kinases
GSK: glycogen synthase kinase ANT: adenine nucleotide translocator
HD: Huntington’s disease
Drp1: dynamin-related protein 1
CREB: cAMP response element-binding protein
DS: down syndrome
Hsps: heat shock proteins
PKC: protein kinase C
mitoK_ATP_: mitochondrial ATP-sensitive K + channel
PINK1: PTEN-induced kinase 1
PD: Parkinson’s disease
PARL: presenilin-associated rhomboid-like serine protease
MKPs: MAP Kinase Phosphatases
NSCLC: non-small-cell lung cancer
RA: rheumatoid arthritis
Shp2: Src homology 2 domain-containing phosphatase 2
NS: Noonan syndrome
LS: Leopard syndrome
JMML: juvenile myelomonocytic leukemia
MPD: myeloproliferative disorder
PTP: protein tyrosine phosphases
PTEN: phosphatase and tensin homolog deleted on chromosome 10
I/R: ischemia/reperfusion
TOM: translocase of the outer membrane of mitochondria
TIM: translocase of inner mitochondrial membrane
MIA: mitochondrial intermembrane space assembly machinery
PAM: presequence translocase-associated motor
SAM: sorting and assembly machinery
